# Temporal Expression of Myogenic Regulatory Genes in Different Chicken Breeds during Embryonic Development

**DOI:** 10.3390/ijms231710115

**Published:** 2022-09-04

**Authors:** Shuang Gu, Chaoliang Wen, Junying Li, Honghong Liu, Qiang Huang, Jiangxia Zheng, Congjiao Sun, Ning Yang

**Affiliations:** National Engineering Laboratory for Animal Breeding and Key Laboratory of Animal Genetics, Breeding and Reproduction, Ministry of Agriculture and Rural Affairs, China Agricultural University, Beijing 100193, China

**Keywords:** chicken, embryonic period, gene expression, myoblast fusion, muscle development

## Abstract

The basic units of skeletal muscle in all vertebrates are multinucleate myofibers, which are formed from the fusion of mononuclear myoblasts during the embryonic period. In order to understand the regulation of embryonic muscle development, we selected four chicken breeds, namely, Cornish (CN), White Plymouth Rock (WPR), White Leghorn (WL), and Beijing-You Chicken (BYC), for evaluation of their temporal expression patterns of known key regulatory genes (*Myomaker*, *MYOD*, and *MSTN*) during pectoral muscle (PM) and thigh muscle (TM) development. The highest expression level of *Myomaker* occurred from embryonic days E13 to E15 for all breeds, indicating that it was the crucial stage of myoblast fusion. Interestingly, the fast-growing CN showed the highest gene expression level of *Myomaker* during the crucial stage. The *MYOD* gene expression at D1 was much higher, implying that *MYOD* might have an important role after hatching. Histomorphology of PM and TM suggested that the myofibers was largely complete at E17, which was speculated to have occurred because of the expression increase in *MSTN* and the expression decrease in *Myomaker*. Our research contributes to lay a foundation for the study of myofiber development during the embryonic period in different chicken breeds.

## 1. Introduction

The major component of the vertebrate carcass is skeletal muscle, which accounts for ~40% of body mass [1]. The development and growth of skeletal muscle are complex, dynamic processes that begin with the proliferation and differentiation of progenitor cells that arise from the mesoderm [2]. Skeletal muscle is composed of bundles of myofibers. A critical event in myogenesis is the fusion of myoblasts either with one another to generate new multinucleated myofibers (hyperplasia) or with an existing myofiber, thereby increasing the myonuclear (hypertrophy) pool and allowing muscle growth [3]. These processes are controlled, through a series of steps, by the regulation of gene expression and posttranslational modification.

Millay et al. [4] identified the muscle-specific membrane protein *Myomaker*, which controls myoblast fusion in the early embryonic development of mice. Several recent studies detected the gene expression of *Myomaker* in the skeletal muscle of adult mice and showed that the gene expression level was low, but the gene was reactivated during myofiber repair after muscle injury, which indicated that *Myomaker* is necessary for myoblast fusion in skeletal muscle growth and development [5,6]. The role of the *Myomaker* gene in muscle development has also been illustrated in zebrafish [7]. In contrast, the function of *Myomaker* in chicken is lagging behind that in other model species. Luo et al. [8] published one of the first studies and confirmed that *Myomaker* was a muscle-specific gene in chickens. The functional verification of *Myomaker* has also been studied in primary chicken myoblasts by overexpression and knockdown of *Myomaker*, and the results showed that the expression of *Myomaker* could promote the fusion of chicken myoblasts [8].

In addition, numerous genetic screens performed in mice, *Drosophila*, and zebrafish have demonstrated that myogenic regulatory factors are involved in myoblast fusion. Members of this gene family, such as *Myf5*, *MYOD*, *MYOG*, and *MRF4*, constitute an interactive regulatory transcriptional network that controls the determination and terminal differentiation of myoblasts [9]. Among these genes, *MYOD* is considered to act as one of the determinants [10]. *MYOD* is essential for both embryonic and adult skeletal muscle growth, and it both induces transcription and promotes myogenesis in embryonic skeletal muscle and is committed to determining muscle plasticity in adult skeletal muscle [11]. *MYOD* can specifically recognize DNA sequences and coordinate myogenic gene expression by binding to a palindromic E-box motif (5′-CANNTG-3′) [12]. During the early differentiation of primary chicken myoblasts, *MYOD* binds to the E-box1 of *Myomaker* to promote the regulation of the promoter and induce the transcription and regional histone modification of *Myomaker*, thereby facilitating the formation of myofibers [8].

The gene function of *MSTN* is distinctly different from that of *Myomaker* and *MYOD*. Double muscling in animals refers to marked hypertrophy of muscle, which more often occurs in cattle, sheep, and pigs. Kambadur et al. [13] conducted a sequence analysis of Belgian Blue, Piedmontese, and normal cattle and found mutations in heavy-muscled cattle breeds that inhibited the expression of *MSTN*, which demonstrated the negative regulatory role of *MSTN* in muscle development. Further studies have found that *MSTN* can inhibit myoblast differentiation by blocking genes induced through the Akt/TORC1/p70S6K signaling pathway [14]. Recently, Kim et al. [15] utilized the D10A-Cas9 nickase technique to generate *MSTN*-knockout chickens by primordial germ cells. Compared with wild-type chickens, the *MSTN*-knockout chickens exhibited significantly larger muscle mass and less abdominal fat deposition in pectoral and thigh muscles. However, the degree of skeletal muscle hypertrophy and hyperplasia caused by *MSTN* loss varied with sex and muscle type.

With the increasing demand for animal meat, more studies on the growth and differentiation of skeletal muscle are needed to improve growth rates. An understanding of the regulation of embryonic and postnatal skeletal muscle growth and development is extremely important in this regard. Additionally, the development of poultry muscle and the amelioration of meat quality have been a major focus of breeders. Muscle growth rate differs among the various breeds of chicken; thus, investigating the expression of myogenesis-related genes in various types of chicken could be a breakthrough to regulate muscle development [16]. However, studies investigating the *Myomaker*, *MYOD*, and *MSTN* gene expression profiles during embryonic development among different chicken breeds are largely unclear. Here, we collected pectoral muscle and thigh muscle tissue of four chicken breeds at embryonic days 11, 13, 15, and 17 (E11, E13, E15, and E17) and postnatal day 1 (D1) for the study of gene expression patterns and to characterize the embryonic muscle development of different chicken breeds.

## 2. Results

### 2.1. Temporal Expression of Myogenic Regulatory Genes during Embryonic Development

**Figure 1:**
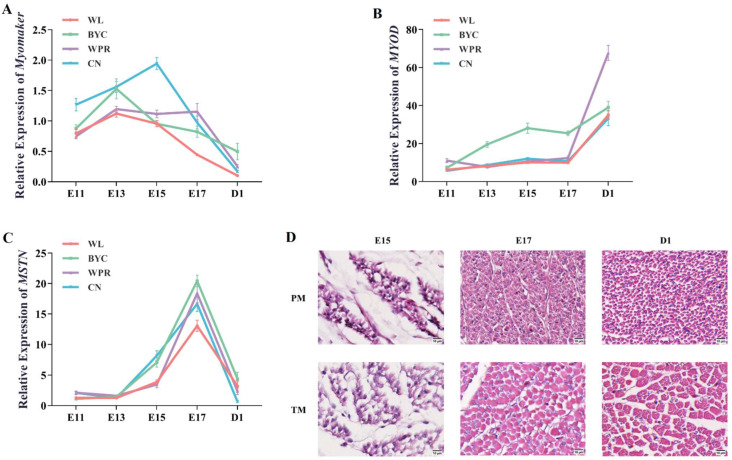
**Time series of the three genes in the late embryonic stage.** (**A**) Expression trends of the *Myomaker* gene in the pectoral and thigh muscles of White Leghorn chicken (WL), Beijing-You Chicken (BYC), White Plymouth Rock (WPR), and Cornish (CN) chickens. N = 24. (**B**) Expression trends of the *MYOD* gene in the pectoral and thigh muscle of WL, BYC, WPR, and CN chickens. N = 24. (**C**) Expression trends of the *MSTN* gene in the pectoral and thigh muscle of WL, BYC, WPR, and CN chickens. N = 24. (**D**) The two columns represent the pectoral muscle (PM) and thigh muscle (TM) of CN chickens. The myoblasts were still fusing at E15, and the contours of myofibers were not clear until E17, indicating that the formation of myofibers had been completed at E17. The diameter of myofibers of TM was obviously larger at D1. Scale bars: 10 μm. For (**A**–**C**) the red, cyan, purple, and blue lines represent changes in the expression levels of the corresponding gene in muscles of WL, BYC, WPR and CN, respectively.

During the embryonic development among the four chicken breeds, the expression levels of the *Myomaker* gene in muscles showed a wave change trend of first increasing and then decreasing (Figure 1A). The *Myomaker* mRNA abundance in both muscle tissues showed an overall increase from E11 to E15 and reached the highest level at E15 of CN and at E13 of the other three breeds (Figure 1A). Afterward, the expression of the *Myomaker* gene across the muscle tissues dropped linearly, although an unusual slight increase was found in WPR at E17 (Figure 1A). Notably, the expression level of the *Myomaker* gene was nearly zero at D1 (except for BYC), due to the decrease in myoblast fusion and the basic formation of myofibers during the late embryonic stage (Figure 1D).

The expression of the *MYOD* gene in muscles of the four chicken breeds was clearly lower during the embryonic stage compared with D1 (Figure 1B). In brief, the gene expression level increased from E11 to E15, decreased slightly from E15 to E17 (*p* = 0.65), and then increased sharply from E17 to D1 (Figure 1B). Unlike that of the previous two genes, the expression level of *MSTN* in all breeds increased greatly with embryonic development and peaked at E17 (Figure 1C). After the chickens hatched, the gene expression level of *MSTN* significantly declined, especially in CN, in which it decreased to nearly zero (Figure 1C).

The fusion of myoblasts increased from E13 to E15 due to the highest gene expression level of *Myomaker*. This result was further corroborated by analyzing the myofiber morphology of chicken embryos (Figure 1D). Histological sections of the myofibers at E15, E17, and D1 were generated, and it could be seen that the myoblasts at E15 were in the process of rapid fusion, which was consistent with the gene expression characteristics of *Myomaker* (Figure 1D). The contours of the myofibers were not clear until E17, indicating that the formation of the myofibers had been completed at E17, after which myofiber hypertrophy was observed (Figure 1D).

### 2.2. Meat-Type Chicken Breed Had the Highest Gene Expression of Myomaker at the Crucial Period

**Figure 2:**
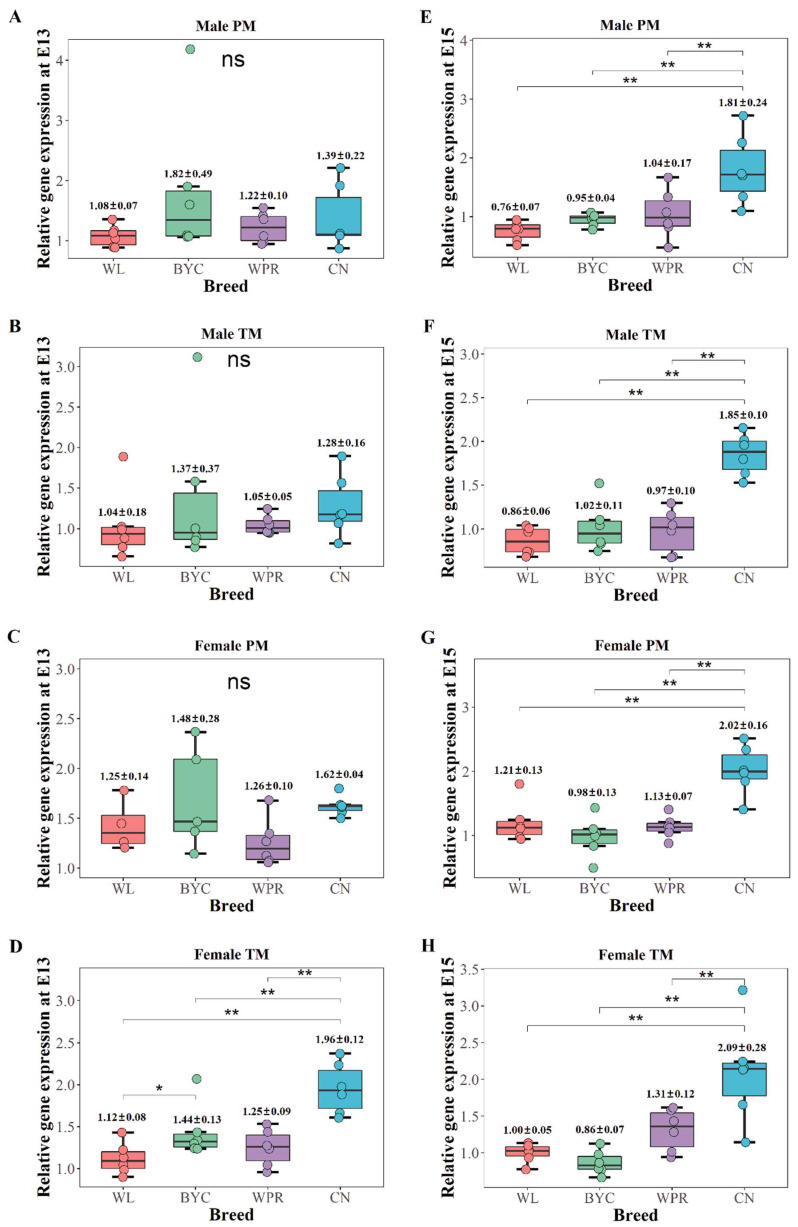
**Gene expression of *Myomaker* among the various chicken breeds.** (**A**–**D**) *Myomaker* gene expression differences among the four chicken breeds in the pectoral muscle of males (PMM), the thigh muscle of males (TMM), the pectoral muscle of females (PMF), and the thigh muscle of females (TMF) at E13. (**E**–**H**) *Myomaker* gene expression differences among the four chicken breeds in the PMM, TMM, PMF, and TMF at E15. **, *, and ns represent adjusted *p* values of <0.01, <0.05, and >0.05, respectively.

Since the crucial period of myoblast fusion was E13 to E15, we further analyzed the differential gene expression of *Myomaker* among various chicken breeds. WL and BYC are the egg-type chicken and native breeds, respectively. CN and WPR are the paternal and maternal lines of modern commercial broilers, respectively. There was no obvious difference in *Myomaker* gene expression among the males of the four chicken breeds at E13 (Figure 2A,B). However, the muscles of CN females had higher *Myomaker* gene expression than those of the other three breeds at E13 (Figure 2C,D). The relative expression of *Myomaker* in the pectoral muscle in CN males at E15 was 1.81 ± 0.24, which was significantly higher than that in the WL, BYC, and WPR males (*p* < 0.01, Figure 2E). Similar results were found in the thigh muscle of males (Figure 2F) and both muscle tissues of the females (Figure 2G,H). These results indicate that the *Myomaker* gene expression in CN was significantly higher than that in the other three chicken breeds at the crucial period, and that the data were robust.

### 2.3. Various Gene Expression of MYOD among the Four Chicken Breeds at D1

**Figure 3:**
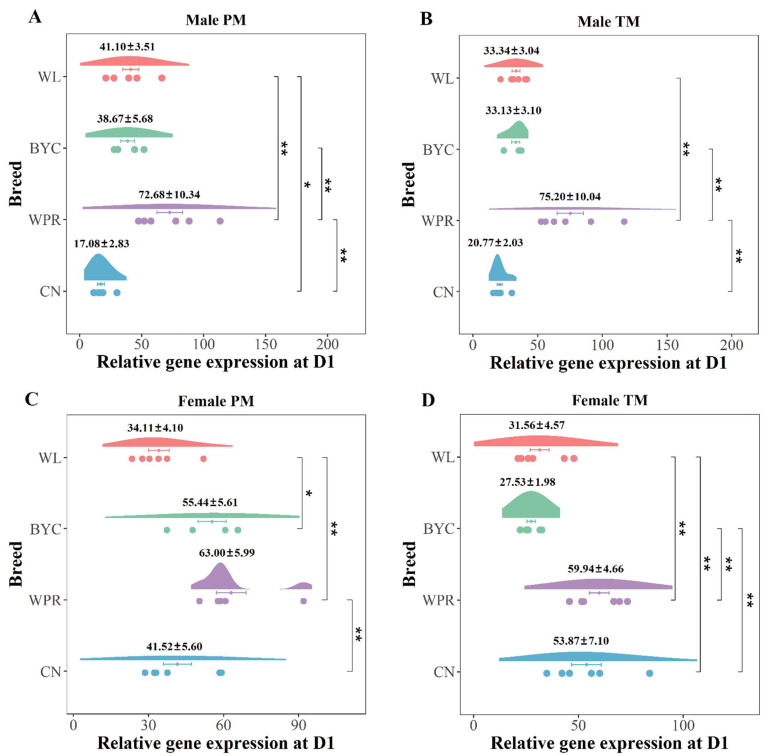
**Gene expression of *MYOD* among the various chicken breeds at D1.** (**A**) Gene expression differences of *MYOD* in the PMM among the four chicken breeds at D1. (**B**) Gene expression differences of *MYOD* in the TMM among the four chicken breeds at D1. (**C**) Gene expression differences of *MYOD* in the PMF among the four chicken breeds at D1. (**D**) Gene expression differences of *MYOD* in the TMF among the four chicken breeds at D1. ** and * represent adjusted *p* values < 0.01 and <0.05, respectively.

As noted above, the expression of the *MYOD* gene in muscles at D1 was higher than that during the embryonic period, which showed little change in *MYOD* gene expression. This result suggested that the *MYOD* gene may more strongly regulate myogenesis after hatching. Therefore, the important time point of D1 was selected to investigate the differential gene expression of *MYOD* among various chicken breeds. There was an extremely significant difference in *MYOD* gene expression in the pectoral muscle (72.68 ± 10.34) and thigh muscle (75.20 ± 10.04) of males between WPR and the other three chicken breeds at D1 (*p* < 0.01, Figure 3A,B). In addition, *MYOD* gene expression in the muscles of males of CN was significantly lower than that in WPR at D1 (*p* < 0.01, Figure 3A,B). The pectoral muscle and thigh muscle of females of WPR still had the highest *MYOD* gene expression among the four chicken breeds at D1 (Figure 3C,D). The difference in gene expression between CN females and the other chicken breed had changed compared to that of CN males at D1. In particular, the *MYOD* gene expression in the thigh muscle of CN females was significantly higher than that in egg-type chickens and Chinese native breeds (*p* < 0.01, Figure 3D), which implied that the gene expression patterns of *MYOD* were slightly different between males and females at D1.

### 2.4. Inhibition of MSTN for the Myoblast Fusion

**Figure 4:**
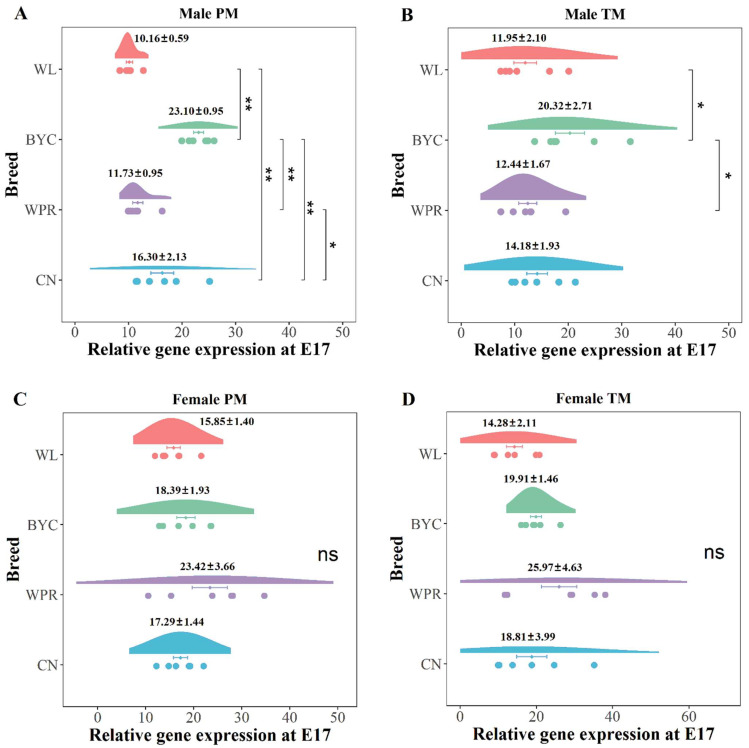
**Gene expression of *MSTN* among various chicken breeds at E17.** (**A**) Gene expression differences of *MSTN* in the PMM among the four chicken breeds at E17. (**B**) Gene expression differences of *MSTN* in the TMM among the four chicken breeds at E17. (**C**) Gene expression differences of *MSTN* in the PMF among the four chicken breeds at E17. (**D**) Gene expression differences of *MSTN* in the TMF among the four chicken breeds at E17. **, *, and ns represent adjusted *p* values < 0.01, <0.05, and >0.05, respectively.

The expression level of the *MSTN* gene was low during the critical period of myoblast fusion, while it reached the highest level at E17, at which time the myofiber development of chicken embryos was repressed. The gene expression of *MSTN* in the pectoral muscle of BYC males (23.10 ± 0.95) was significantly higher than that of WL and WPR males at E17 (*p* < 0.01, Figure 4A). Similar results were obtained in the thigh muscle of BYC males (Figure 4B). Interestingly, there was no significant difference in *MSTN* in the muscles of females among various chicken breeds at E17 (Figure 4C,D), which indicated that *MSTN* gene expression in muscles might be different between male and female chicken embryos.

## 3. Discussion

Our findings demonstrated that E13 to E15 was the crucial period of myoblast fusion in chicken embryos and that *Myomaker* gene expression in CN was the highest. The *Myomaker* gene is closely related to the fusion of myoblasts to myofibers during the embryonic stage [17]. *Myomaker* gene expression decreased after the crucial period, which implied that the process of myofiber formation was essentially complete. This hypothesis was confirmed by the increase in *MSTN* gene expression at E17. Since the *MYOD* gene expression of D1 in muscles was significantly higher than that in the embryonic period, we thought *MYOD* was more important in myofiber hypertrophy after the chicken hatched.

The *Myomaker* gene is closely related to the fusion of myoblasts to myofibers during the embryonic stage [17]. Like in a previous study [8], *Myomaker* gene expression was highest at E14 but sharply decreased after E16. We found that E13 to E15 was associated with the highest expression of *Myomaker* among the four different types of chicken breeds, suggesting that E13 to E15 was considered a critical stage in myofiber development. The increase in *MSTN* gene expression at E17, together with the decrease in *Myomaker* gene expression, implied that myoblast fusion and muscle development events were inhibited from E17 to D1, which in turn proved the occurrence of atrophy of skeletal muscle in late-term chicken embryos [18]. The atrophy of skeletal muscle was associated with the rapid growth of the intestinal tract and the inhibition of protein synthesis in the liver, leading to the catabolism of protein in skeletal muscle to provide energy for chicken embryos [19,20]. Our results showed that the *Myomaker* gene was barely expressed in muscles at D1, which verified previous the findings of studies showing that muscle morphology formation occurred during embryogenesis [21].

We found that *MYOD* gene expression in muscles at D1 was much higher than that during the embryonic period, which was consistent with the findings of a previous study [8]. Unlike the results in chickens, *MYOD* gene expression in mice increased until birth, at which point the expression decreased [22]. This finding indicated a difference in *MYOD* gene expression between vertebrates and mammals during the embryonic period. Osamu Saitoh et al. [23] studied the expression of myogenic genes in pectoral muscle of chicken in the late embryonic stage and post-hatch and found that *MYOD* expression decreased steadily during late embryogenesis but showed a resurgence in postnatal day 2. This is a little different from our result, which was that the expression level of *MYOD* increased smoothly in the late embryonic period. Our study found *MYOD* gene expression differences in males and females between broilers and laying hens and local chickens. The results suggested that there might be breed variations in the *MYOD* gene expression pattern in chickens. The expression pattern of the *MYOD* gene might be affected by the process of selection between chicken breeds. As an important gene to activate and promote skeletal muscle development, there is little research on the gene expression differences of *MYOD* between different breeds. Evidently, more research on the difference of gene expression among varieties and regulatory mechanisms can be carried out in the future.

Studies have found that the *MSTN* gene can inhibit the activity and expression of the *MYOD* gene, thereby controlling the differentiation of myoblasts into myotubes, and that this inhibition is mediated through the Smad3 pathway [24]. The 55% reduction in *MSTN* expression along with the up-regulation of *MYOD* by 4.65 times in chicken embryonic myoblasts further demonstrated the negative correlation between the two genes [25]. Nonetheless, relatively little research has been conducted on the expression patterns of the *MSTN* gene during the embryonic period of chickens. In the present study, *MSTN* gene expression in both the pectoral muscle and thigh muscle increased from E11 to E17 in all four chicken breeds, which was consistent with the results for other organs, such as the liver, heart, brain, and intestine [26]. As the chicken embryo develops to E17, the nutrients in amniotic fluid are used for the functional development of the gastrointestinal tract, and the morphological changes of the intestinal mucosa are accelerated [19]. The gene expression of *MSTN* during E15 to E17 increased sharply, suggesting that chicken embryos prepare for feeding after hatching at this stage through the rapid development of the digestive and metabolic organs; therefore, muscle growth in the late embryonic period is temporarily inhibited.

In addition to breed, maternal nutrition may also have an effect on the expression of myogenic genes. Restricted maternal nutrition will reduce the myogenic regulatory factor expression [27]. On the other hand, there were studies that found that adding L-arginine or lactic acid to the diet would increase the gene expression of *MYOD* in muscles, thereby promoting muscle development [28,29]. The *MSTN* gene expression was down-regulated at weaning and up-regulated during the finishing period due to a maternal low-protein diet, indicating that the expression pattern of *MSTN* was stage-specific [30]. Besides, short-term fasting could not elicit the marked alteration of the myogenesis genes regulating muscle development [31]. However, the *MSTN* expression was decreased by underfeeding in rats, and this was a long-term trend [32]. These studies suggest that maternal nutrition also plays an important role in muscle development, and the combination of genetics and nutrition is required in the future to study the muscle development.

E13 to E15 is considered a critical stage of myoblast fusion in embryonic development. The formation of myofibers in various chicken breeds at this possibly critical stage is an important foundation for the growth rates of skeletal muscle in chickens after incubation. The differences between broilers and laying hens have increased due to the intensive genetic selection of modern breeding for important traits [33]. Selection effects are often detected in chicken embryos, which are excellent models for development mechanisms [34,35]. Previous studies have shown that the body weight and pectoral muscle weight of broilers were larger than those of laying hens, from E11 to E18 [36]. The increased proliferation and differentiation activity of myoblasts, increased number of nuclei in myofibers, and increased diameter of myofibers at E15 also proved that broilers grow faster during the embryonic stage. We found that the expression of the *Myomaker* gene in the muscles of CN was the highest at the critical stage of myoblast fusion and was significantly higher than that of other chicken breeds at E15. These results implied that the myoblasts fused to myofibers more in CN, a cornerstone broiler breed that develops rapidly, than in other chicken breeds. This might be the reason why fast-growing broilers such as CN chickens had greater muscle mass a few weeks after hatching.

## 4. Materials and Methods

### 4.1. Bird and Sample Collection

White Leghorn (WL) is an egg-type chicken breed with a slow growth rate and excellent laying performance. Beijing-You Chicken (BYC) is a native Chinese breed with good meat quality. Cornish (CN) and White Plymouth Rock (WPR) are two popular breeds due to their rapid growth rate and have been extensively used worldwide as the paternal and maternal lines of commercial broilers, respectively. The fertile eggs of WL and BYC were obtained from the Poultry Genetic Resource and Breeding Experimental Unit of China Agricultural University. The fertile eggs of WPR and CN were provided by Beijing Huadu Yukou Poultry Industry Co., Ltd. (Beijing, China). All fertile eggs were sterilized with Benzalkonium bromide solution before incubation. The temperature and humidity in the incubator were maintained at 37.8 °C and 60%, respectively.

**Figure 5:**
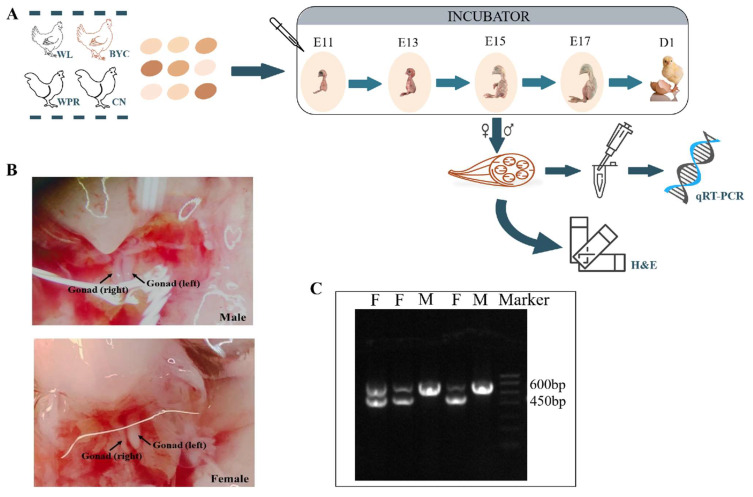
**Experimental design and sex determination.** (**A**) Experimental design. In this study, four chicken breeds and five embryonic important time points were selected to explore the expression levels of three muscle-related genes in pectoral muscle and thigh muscle. In addition, the H&E slices were made for the observation of the histomorphology of myofibers. (**B**) Graphical representation of different sexes. For males, both sides of the gonads were the same size; for females, the left side of the gonads was larger than the right side. (**C**) Reconfirmation of sexes by gel mapping. For males (M), one band (600 bp) was visible; for females (F), two bands (600 bp and 450 bp) were visible.

The overall flow of the present study is shown in Figure 5A. More than 40 fertile eggs were randomly examined for the presence of viable embryos at each embryonic timepoint (E11, E13, E15, and E17). The viable embryos were sacrificed by decapitation for subsequent experiments. The remaining fertile eggs were left in the incubator until the chicks hatched. After hatching, more than 20 healthy chicks were killed by cervical dislocation. The right pectoral muscle and thigh muscle, which were trimmed free of fat, were collected from each chicken embryo. There are 6 biological replicates for each gender and group. All muscle tissues were immediately saved in RNase-free tubes containing RNAlater and stored at −20 °C until RNA extraction. All experiments with chicken in this study were approved by the guidance of ethical regulations from the Laboratory Animal Welfare and Animal Experiment Ethics Committee of China Agricultural University (Approval Code: AW71802202-1-2).

### 4.2. Sex Determination

Sex was first judged by gonadal development after the abdomen was opened. In brief, both sides of the gonads being the same size indicated males, while a left side being larger than the right side indicated females (Figure 5B). To reconfirm the sex of the chicken embryos, a PCR amplification reaction of *CHD1* genes located on the Z chromosome was conducted [37]. The males were identified by the presence of one band (600 bp) after agarose gel electrophoresis, while the females were identified by the presence of two bands (600 bp and 450 bp) (Figure 5C). After sex determination, six males and six females collected at each time point were used for the subsequent analysis.

### 4.3. Total RNA Extraction and Cdna Synthesis

Approximately 20 mg of muscle tissue was transferred to a 1.5 mL tube (Axygen, Silicon Valley, CA, USA) with 300 μL of lysis reagent (Promega, Madison, WI, USA) and then homogenized three times at 65 Hz for 30 s each. Then, 300 μL of RNA dilution liquid was added to the homogenate and incubated for 5 min until centrifugation at room temperature at 13,000 rpm, and total RNA was extracted, in accordance with the protocol of the manufacturer (Promega). The RNA concentration was detected by a NanoDrop spectrophotometer (Thermo, Waltham, MA, USA), and the quality was assessed by agarose gel electrophoresis. Total RNA was qualified and used for the next manipulation (Figure S1). cDNA was synthesized using the PrimeScript RT Reagent Kit with gDNA Eraser (Takara, Shiga, Japan) in a 10 μL volume, in accordance with the instructions of the manufacturer.

### 4.4. Quantitative Real-Time PCR

Two microliters of cDNA were added to 18 μL of PCR master mix containing 10 μL TB Green Premix Ex Taq (Takara, Shiga, Japan), 2 μL of ROX Reference Dye II (Takara, Shiga, Japan), 0.8 μL of forward primer (10 μM), 0.8 μL of reverse primer (10 μM), and 6 μL of sterilized water. The primer sequences for target genes, which included *Myomaker*, *MYOD*, *MSTN*, and the internal reference gene (*β-actin*), are listed in Table 1. The reactions were run on an Applied Biosystems 7500 real-time PCR instrument using the following settings: 95 °C for 30 s, 40 cycles of 95 °C for 5 s, and 60 °C for 34 s; and a dissociation step consisting of 95 °C for 15 s, 60 °C for 1 min, and 95 °C for 15 s. For all qPCR analyses, three technical replicates were included for each sample. Relative expression was calculated with *β-actin* as a housekeeping gene, using the 2^−ΔΔCt^ method [38]. Changes in gene expression were calculated separately for pectoral muscle and thigh muscle using the average ΔCt value of *Myomaker* for pectoral muscle of WL males at E11 as the control.

### 4.5. Hematoxylin-Eosin (HE) Staining

The pectoral muscle and thigh muscle samples were carefully collected from E15 to D1 chicken embryos and fixed in 4% paraformaldehyde for more than 48 h. The samples were dehydrated in 20%, 30%, 40%, 50%, 60%, 70%, 80%, 90%, and 95% ethanol solutions successively and then embedded in paraffin wax. The transverse sections of 3 μm were cut and stained with hematoxylin and eosin for morphology examination [39].

### 4.6. Statistical Analysis

The results are expressed as the means ± SEs of six independent biological replicates. Statistical analyses were performed using one-way analysis of variance followed by least significant difference (LSD) tests via the R program (ver 3.6.1). *p* values of less than 0.05 and 0.01 were considered to be significant and very significant, respectively.

## 5. Conclusions

This study reported the dynamic expression of key genes that influence muscle development in the late stage of chicken embryos. The expression of genes in the pectoral and thigh muscles could be controlled by a shared myogenic regulatory program. We found that E13 to E15 was the critical period of myoblast fusion and contributed significantly to the rapid growth of broilers. Since the increase in *MSTN* gene expression occurred together with the decrease in *Myomaker* gene expression at E17, we speculate that the formation of myofibers is nearly complete at E17 and that the process of myofiber hypertrophy begins after that. There might be breed variations in the *MYOD* gene expression pattern in chickens, since differences in gene expression were detected among meat-type, egg-type, and native chickens between males and females at D1. Our research also lays a foundation for the study of myofiber development during the embryonic period in different chicken breeds.

## Figures and Tables

**Table 1:** The RT-qPCR primer sequence of genes.

Primer Name	Primer Sequence (5′-3′)	Annealing Temperature (°C)
*CHD1*	F-GTTACTGATTCGTCTACGAGA	52
	R-ATTGAAATGATCCAGTGCTTG	
*β-actin*	F-ATCTTTCTTGGGTATGGAGTC	60
	R-GCCAGGGTACATTGTGG	
*Myomaker*	F-TGGGTGTCCCTGATGGC	60
	R-CCCGATGGGTCCTGAGTAG	
*MYOD*	F-GCTACTACACGGAATCACCAAAT	60
	R-CTGGGCTCCACTGTCACTCA	
*MSTN*	F-ACAGTAGCGATGGCTCTTT	60
	R-CCGTTGTAGGTTTTTGGAC	

*CHD1*: chromodomain helicase DNA binding protein 1; *β-actin*: actin, beta; *Myomaker*: myomaker, myoblast fusion factor; *MYOD*: myogenic differentiation 1; *MSTN*: myostatin.

## Data Availability

The data presented in this study are available in the Supplementary materials.

## Supplementary Materials

The supporting information can be downloaded at: https://www.mdpi.com/article/10.3390/ijms231710115/s1.

## Abbreviations

WL	White Leghorn
BYC	Beijing-You Chicken
WPR	White Plymouth Rock
CN	Cornish
PM	Pectoral muscle
TM	Thigh muscle
PMM	Pectoral muscle of males
PMF	Pectoral muscle of females
TMM	Thigh muscle of males
TMF	Thigh muscle of females
