# Supplementary figures and images for: Temporal Expression of Myogenic Regulatory Genes in Different Chicken Breeds during Embryonic Development
Source: Int J Mol Sci. 2022 Sep 4;23(17):10115. doi: 10.3390/ijms231710115 (PMC9456251; doi:10.3390/ijms231710115)

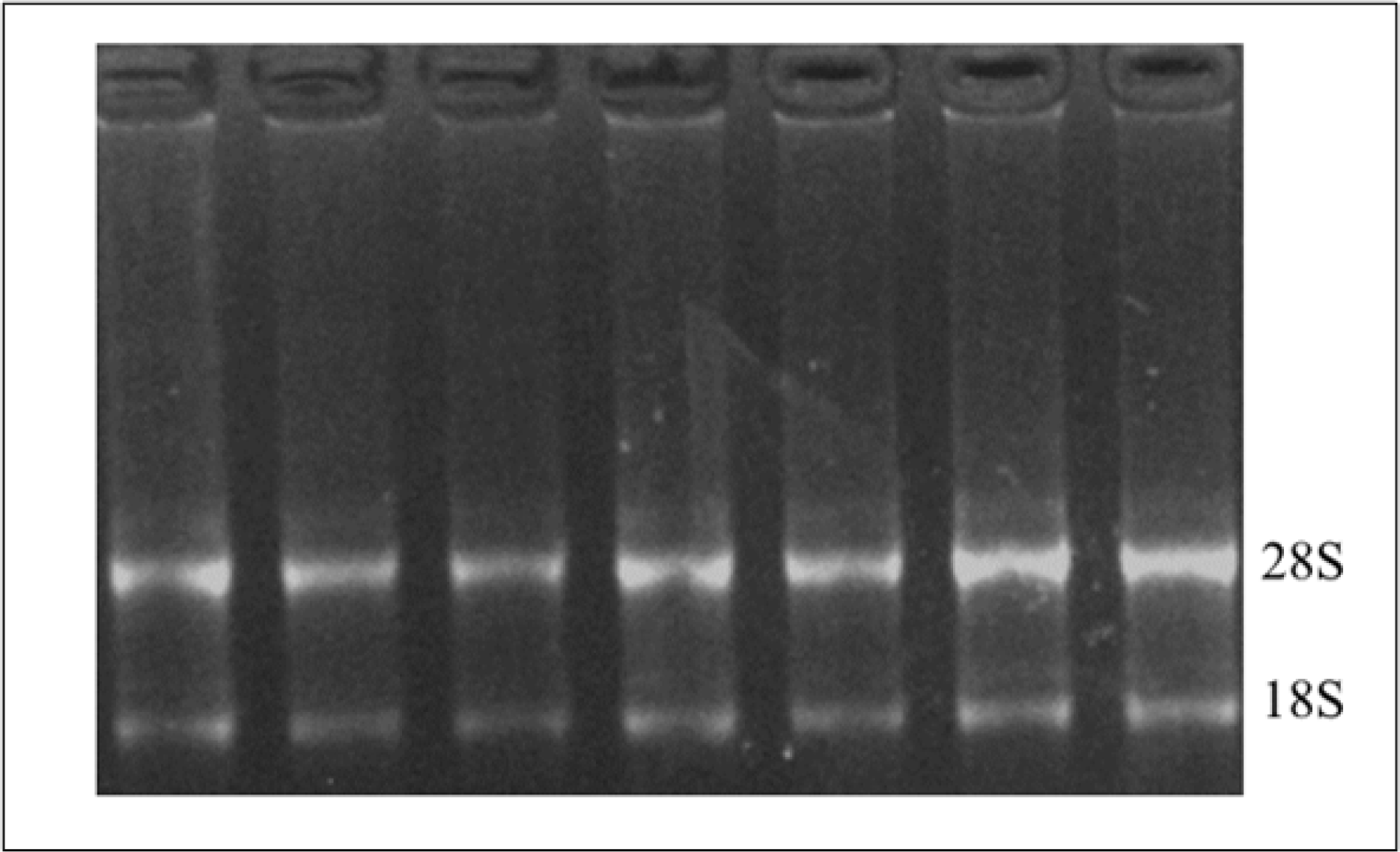

Supplement: Supplementary file 1 [file ijms-23-10115-s001.zip › Figure S1.tif]
